# Exploring the Linkage between the Neighborhood Environment and Mental Health in Guangzhou, China

**DOI:** 10.3390/ijerph16173206

**Published:** 2019-09-02

**Authors:** Yingzhi Qiu, Yuqi Liu, Yi Liu, Zhigang Li

**Affiliations:** 1 College of Urban and Environmental Sciences, Peking University, Beijing 100871, China; 2 Department of Urban Planning and Design, The University of Hong Kong, Hong Kong 999077, China; 3 Institute of Planning and Design, Anhui Institute of Urban Construction and Design, Hefei 230051, China; 4 School of Urban Design, Wuhan University, Wuhan 430072, China; 5 Institute of Hubei Human Habitat Engineering and Technology, Wuhan 430072, China

**Keywords:** built-environment, social environment, mental health, neighborhood boundary, China

## Abstract

The relationship between the neighborhood environment and mental health has been investigated mostly in developed countries. Yet few studies have systematically examined the impact of the neighborhood-level built-environment and social environment on mental health within different localities in the Chinese context. Based on a household survey and geographical data in Guangzhou, China, this study aimed to explore the linkage between the neighborhood environment and mental health, with a particular focus on aspects of the built-environment that are related to new urbanism or compact cities and contextual social capital, using three geographic delineations. Our findings indicated that built-environment indicators based on a road network buffer had a higher explanatory power towards residents’ mental health than did those based on a circular buffer. The analytical models demonstrated that neighborhood floor-area ratio, building density, and per capita green area were positively correlated with mental health. Neighborhood safety and contextual neighborhood interactions and reciprocity had positive associations with mental health. These findings provide policy makers and urban planners with valuable information on the role of the compact city strategy and the neighborhood social environment to improve the mental health of residents.

## 1. Introduction

Mental health refers to a state of well-being in which an individual realizes that his or her own abilities can cope with the normal stresses of life, work productively and fruitfully, and make a contribution to his or her community [1]. In recent years, the relationship between the urban environment and residents’ mental health has become an emerging multidisciplinary research field of health geography, environmental science, public health, psychology, urban and rural planning, and sociology [2,3,4]. Various empirical studies conducted in western countries have reported significant impacts of neighborhood environmental characteristics on residents’ mental health, such as housing conditions, greenspace, neighborhood social support, etc. [5,6,7]. People from developing countries are facing greater risks of mental disorders, due to severe poverty, inadequate health facilities, rapid urbanization, and more exposure to environmental hazards [8,9]. In China, problems with mental health have received unprecedented attention from the Chinese government and from residents in recent years, as rapid urbanization has transformed environmental and social conditions in ways that may threaten the mental health of residents [10,11,12]. Exploring the effects of neighborhood environmental characteristics on mental health outcomes has thus become significant for both policy makers and urban planners to improve the mental wellbeing of Chinese residents.

The built-environment and the social environment are two domains of neighborhood context that are related to mental health. A plethora of studies have documented a positive relationship between greenspace and mental health [13,14,15]. The mechanism can be summarized into the following three pathways. First, greenspace is recognized as a resource for psychological restoration that helps residents to reduce stress and restore attention [7,16]. Second, greenspace is generally one of the preferred choices for residents to conduct physical activity, which is likely to be beneficial for psychological health [17,18]. Last but not least, greenspace provides a public place for residents’ daily social interactions, such as chats and group activities, which enhances social capital within the neighborhood and thereby produces psychological benefits [19]. Besides greenspace, housing conditions and service facilities can also affect mental health status [6,20,21]. Since the concepts of new urbanism and compact cities, often characterized by relatively high density, high walkability, and high land use efficiency, have been promoted for the renovation and construction of neighborhoods, the relationship between environmental attributes related to such concepts and health outcomes has received increasing attention. Empirical studies have found that high neighborhood walkability and accessible facilities are beneficial to mental health [22,23,24].

The delineation of a neighborhood environment that may affect residents’ mental health is important in quantitative analysis. Methods used in existing research can be classified into three categories. First, the neighborhood environment is strictly enclosed within one neighborhood, defined by a census, postal sector, or administrative boundary [21,25,26]. Given that the residents’ daily environmental exposure is not just constrained within the neighborhood, the second approach defines the boundary as a neighborhood-centric buffer zone with radii of varying sizes, ranging from 100 m to 3 km [27,28]. The third method concerns the proximity or accessibility of certain spaces that influence mental health, and thus uses distance or time as the main indicator [29,30]. However, the selection of a built-environmental impact area may lead to significant measurement error because of the uncertain geographic context problem (UGCoP). The UGCoP occurs when the environment measured in the research deviates from the actual geographic context to which an individual is exposed in daily life [31]. This problem is likely to result in the inconsistent findings regarding the association between contextual environment and health outcomes. Most existing studies only use one specific geographic delineation method, and rarely examine the “true causally relevant” geographic context [31,32]. For example, different delineations of the neighborhood lead to different and even conflicting results of the relationship between the greenspace and health [33]. Given UGCoP when using the geographic buffer method, the circular buffer might be less appropriate, as some areas that included are inaccessible to residents. To overcome such problems, employing the road network buffer based on street network and walk time can better capture the characteristics of the neighborhood’s built-environment, where residents get access to in daily life [13,34]. This method is verified to be more accurately representative of the land use characteristics than circular buffers [35]. However, regarding to the mental health, previous studies failed to address which geographic delineation can better interpret the impact of neighborhood environment on individuals’ mental health.

Neighborhood social environment is recognized as an important dimension that affects mental health. Evidence shows a significant positive association between the socio-economic status (SES) of a neighborhood and mental health after controlling for personal socio-demographic attributes [36,37]. Stressors inside socio-economically deprived neighborhoods, such as anti-social behavior or environmental disorder, stimulate negative emotions and even stress, thus making residents more likely to have higher levels of common mental disorders [38]. Social capital, generally defined as features of social organizations, such as reciprocity, trust, and civic engagement [39], is another important neighborhood determining factor [5]. Neighborhood reciprocity and trust refer to the extent to which residents are willing to help and trust each other respectively [39,40]. Civic engagement refers to the level of residents’ involvement in their neighborhoods [40]. It is important to distinguish between individual-level (compositional) social capital and neighborhood-level (contextual) social capital. Individual-level social capital may influence mental health through social influence, social engagement, and social support [40,41,42]. Neighborhood-level social capital affects mental health through different pathways, such as collective socialization, informal social control, and collective efficacy [39,41]. In addition, neighborhood safety has been proven to have an influence on residents’ mental health [43,44]. Crimes and anti-social behaviors in neighborhoods can directly put pressure on residents [45]. Social disorder and problems of unsafe neighborhoods can negatively influence residents’ mental health [46].

In the Chinese urban context, an increasing number of studies have investigated the associations between the neighborhood environment and mental health outcomes in recent years. As one of the most important characteristics of neighborhood environment, greenspace is positively related to mental health through direct impacts and indirect pathways [15,47,48]. Housing conditions, such as living area, housing quality and housing type, also significantly affect mental health [49]. Street walkability, accessibility of facilities and amenities within neighborhoods are positively associated with mental wellbeing of mid-aged and old people [21,22,23,50]. Neighborhood social attributes, including individual-level social capital and perceptions of neighborhood safety are found to be determinants of residents’ mental health [29,51]. However, several problems are still not fully addressed. First, some built-environment factors related to concepts of new urbanism and compact cities are not sufficiently examined in existing literature. Such concepts have been strongly advocated as a strategy to improve the quality of living space and achieve sustainable development in Chinese cities with high population densities [52]. Nevertheless, the relationship between building density, floor area ratio, and mental health is poorly understood. Second, few studies have improved measurement techniques to solve the UGCoP. It is still unclear which measurement can most accurately reflect the impact of the neighborhood environment on mental wellbeing. Third, regarding the neighborhood social environment, most prior studies have focused on compositional social capital, with only a few exploring the relationship between contextual social capital and mental health in adolescents and elders [53,54], not involving all age groups.

To fill in the above knowledge gaps, this study aims to systematically explore the impact of a neighborhood’s built-environment and social environment on mental health in the Chinese context. We focus on two particular aspects of the built-environment that are related to so-called new urbanism, the compact city and contextual social capital, which have not been sufficiently discussed. Furthermore, this study contributes to the literature by measuring individuals’ more realistic exposure to the geographic context. We hypothesize that greenspace, high density, neighborhood social capital, and neighborhood safety are positively related to mental health.

## 2. Data and Methods

### 2.1. Data and Research Area

**Figure 1:**
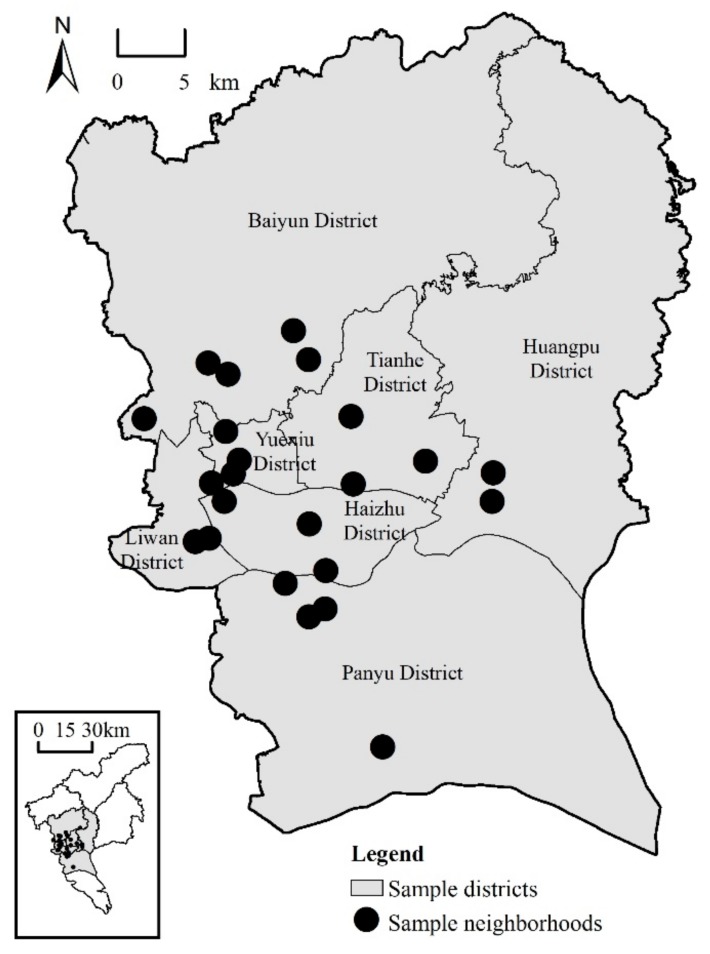
Location of 23 sampled neighborhoods in Ghuangzhou, China.

The data analyzed in this study were derived from a questionnaire survey, GIS spatial data, and census data. For the questionnaire survey, we conducted a household survey in Guangzhou, China in 2015. Firstly, a multi-stage stratified probability proportionate to size (PPS) sampling method was used to randomly select 23 neighborhoods from 7 districts located in inner city areas and inner suburbs (Figure 1). Secondly, based on house number, around 50 households were randomly selected in each sampled neighborhood using a systematic sampling technique. Thirdly, following the Kish selection method, an adult over the age of 18 was chosen in each sampled household to complete the questionnaire. Overall, the survey yielded 1150 valid questionnaires; the return rate was 97.5%. The questionnaire contained demographic information, socioeconomic status, mental health conditions, and social capital.

The GIS spatial data were derived from high resolution Google Earth images using ArcGIS (Esri, Redlands, CA, USA). This dataset included information about road networks, buildings, and greenspace within different buffer zones of each sampled neighborhood. The population in the sampled neighborhoods’ buffer areas was calculated based on data from the sixth census for Guangzhou, which was conducted by the National Bureau of Statistics in 2010.

### 2.2. Delineation of Built-Environment Impact Area

In order to match individuals’ real daily activity space as much as possible, a network buffer was chosen to determine the boundary of the built-environment. The network buffer of a neighborhood was established based on the road network, thus was able to measure the built-environment that people actually access around the neighborhood [55]. The size of the network buffer for each neighborhood was unique, based on the connectivity of the road network. In general, the higher the street connectivity, the larger the network buffer. The reason why we generated a 15-min walking distance network buffer is because many urban neighborhoods in China, such as Shanghai’s, Hangzhou’s, and Changsha’s, have been designed and built with the guidance of a 15-min life circle planning strategy. The 15-min living circle serves as the basic geographic area for residents’ living and daily activities, and requires sufficient infrastructure, public service facilities and public space within 15 minutes’ walking distance. Therefore, based on the road network and two to four entrances to each neighborhood, we generated 23 network buffers with the range of 15 minutes’ walk (the walking speed is 72 m/min, the general maximum walking speed of Chinese people) from each sampled neighborhood using ArcGIS Network Analyst.

**Figure 2:**
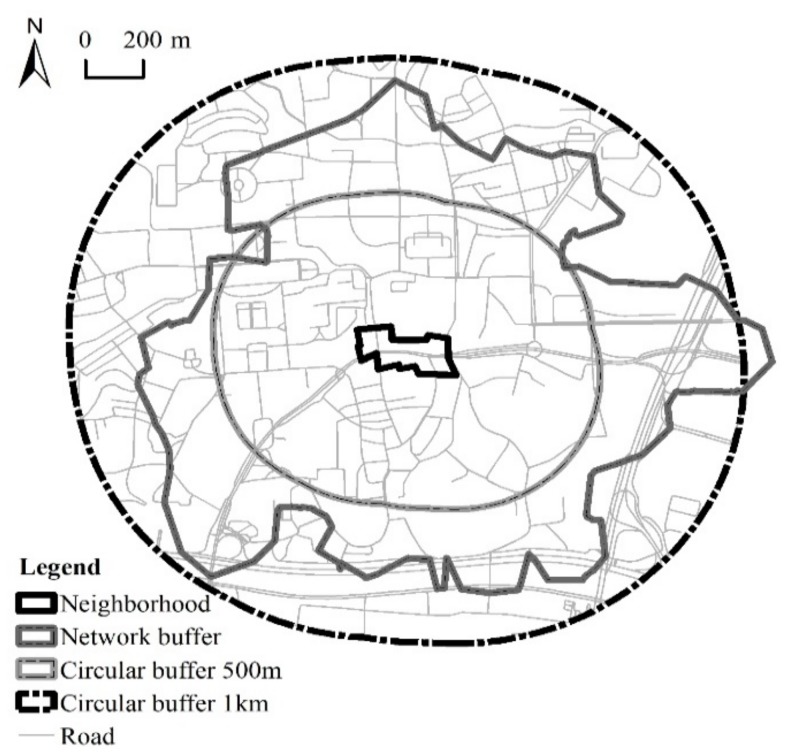
Three different buffer areas of one sampled neighborhood (Yuezhou).

We also adopted another two circular buffers around the neighborhood as comparative built-environment impact areas. To be specific, starting from the neighborhood boundary, two buffers with linear units of 500 m and 1000 m were formed. Figure 2 shows three different buffer areas around one surveyed neighborhood.

### 2.3. Measures of Mental Health

Mental health outcomes were measured using the 12-item General Health Questionnaire (GHQ), which was developed by Goldberg and Blackwell [56]. It is an extensively used instrument designed to screen for nonpsychotic psychiatric distress and has been found to be both valid and reliable for use in China [57]. Comprising 12 items, the GHQ-12 asks the respondents whether they have experienced a particular mood recently with a four-point scale (‘less than usual’, ‘no more than usual’, ‘rather more than usual’, or ‘much more than usual’). The customary scoring methods used are bi-modal method (0-0-1-1) and Likert scoring method (0-1-2-3) [58]. We chose the Likert scoring method for this study, since it contained the most information for the linear regression model (Cronbach’s alpha was 0.80 in this study, indicating good internal consistency). The total GHQ score was generated by adding all the items ranging from 0 to 36. Higher scores indicate poorer mental health [59].

### 2.4. Independent Variables

#### 2.4.1. Built-Environment

Built-environment was captured using three neighborhood level variables. Building density referred to the ratio of the total base area of the buildings in the buffer zone to the total area of the buffer zone. The per capita green area referred to the ratio of the total area of greenspace to the population within the buffer area. Greenspace included residential cluster greenspace, public greenspace, productive plantation area, and protective greenbelt. Population referred to the total number of residents living in the buffer area. Since the buffer area covered more than one neighborhood and the boundaries of some neighborhoods were intersected by the buffer boundary, the land area weight was developed for adjustment. The land area weight of each neighborhood was calculated as the percentage of the neighborhood area within the buffer area relative to the total neighborhood area. The population of each neighborhood within the buffer area was then obtained by multiplying the total population of the neighborhood by the land area weight. Finally, the total population of the buffer area was obtained by summing the neighborhood population calculated above. Floor area ratio was measured only within the neighborhood administration boundary. This is because the floor area ratio directly affects the living comfort of the neighborhood, and is one of the restrictions imposed by urban planning legislation on the neighborhood construction of developers in China. Floor area ratio was measured in terms of the ratio of a building’s total floor area (the area of the building base multiplied by the number of building floors) relative to the total area of the neighborhood.

#### 2.4.2. Social Environment

Neighborhood social capital and safety were two dimensions related to the social environment. The operationalization of neighborhood-level social capital followed the strategy developed by Kawachi and his colleagues [39,40]. Social capital was measured based on respondents’ answers to four questions about neighborhood interaction, participation, reciprocity, and trust. Neighborhood interaction and neighborhood participation were measured in terms of the frequency of respondents’ interaction with neighbors and participation in neighborhood activities respectively, with the answers ranking from 1 (‘never’) to 5 (‘always’). Respondents whose choices were 4 and 5 were considered to have high neighborhood interaction and high neighborhood participation. The proportion of these respondents was taken as the neighborhood-level indicator. Neighborhood reciprocity and neighborhood trust were measured based on whether respondents agreed that ‘‘people are willing to help each other in the neighborhood,” and “if one day I have to leave home for a period of time, I can count on neighbors to help collect express parcels, registered letters, newspapers, etc.,” respectively. The answers were ranked from 1 (‘strongly disagree’) to 5 (‘strongly agree’), and respondents choosing 4 and 5 were seen as having high levels of neighborhood reciprocity and neighborhood trust. Similarly, the proportions of respondents with high neighborhood reciprocity and neighborhood trust were calculated as the neighborhood-level indicators, respectively. Neighborhood safety was operationalized as the number of disputes that occurred in the neighborhood in 2014. Higher numbers of disputes were taken to indicate less safe neighborhoods.

Neighborhood socio-economic status can synthetically reflect the built-environment and social environment to a certain extent, which may cause endogenous problems in the analysis, thus is not discussed in this study.

#### 2.4.3. Socio-Demographics

A series of individual-level predictors were included in the regression analysis; namely, demographic characteristics, socioeconomic status, housing conditions, and hukou status. Three demographic variables were included. Gender was a dummy variable, with female respondents coded 1 and male respondents 0. Age was a continuous variable, ranging from 18 to 67. Marital status and family organization was trichotomized into single/divorced/widowed, married and living with family, and married but not living with family (more common in migrant populations). Socioeconomic status was captured using two variables. Education was categorized into junior high school and below, technical school/high school, and college/university and above. Per capita household income was a continuous variable. Housing conditions were measured using per capita living space and housing tenure. Per capita living space was a continuous variable. Housing tenure was a dummy variable, coded 1 if respondents owned housing tenure, 0 otherwise. Hukou status was dichotomized into Guangzhou hukou holders and others (reference group). Table 1 summarizes the variables used in this study.

### 2.5. Multilevel Model Analysis

Multilevel model has been widely used in public health research because of its ability to explore the contextual and compositional effects on health [26,60,61]. This study employed a multilevel linear model, due to the two-level hierarchical nature of the data (individual-level data and neighborhood-level data) and the continuous variable of GHQ score. The two-level random-intercepts model is specified as:

*Y_ij_* = *α* + *βW_j_* + *γX_ij_* + *u_j_* + *ε_ij_*, (1)

This equation assumes:

E(*ε_ij_*) = 0, Var(*ε_ij_*) = *σ^2^*; E(*u_j_*) = 0, Var(*u_j_*) = *τ^2^*; Cov(*u_j_*, *ε_ij_*) = 0, (2)

In this equation, *i* represents the individual indicator and *j* represents the neighborhood indicator; *Y_ij_* refers to the GHQ score for individual *i* in neighborhood *j*; *γ* is the coefficient of individual variables. *α* and *β* are coefficients of neighborhood variables and are fixed-effect; *X_ij_* refers to a vector of individual variables; *W_j_* refers to a vector of neighborhood variables; *ε_ij_* and *u_j_* represent random error terms at the individual and neighborhood levels, respectively; *σ^2^* and *τ^2^* are variances at the individual and neighborhood levels, respectively.

The models were fitted using the STATA program version 14.0 (StataCorp LP., College Station, TX, USA). Multicollinearity was first detected using the variance inflation factor (VIF). The results showed that the VIF of each predictor variable in the model was less than three, meaning no serious multicollinearity. Then, the null model was constructed to estimate the contribution of different levels of variables in explaining the differences of residents’ mental health in different neighborhoods. Third, four sets of models were initiated to estimate the effects of the neighborhood environment on mental health. Considering that the stable environmental impact of a neighborhood on the mental health of residents took time to establish, the sample of residents who had lived in the neighborhood for less than one year was excluded, leaving a total of 1124 valid samples.

Higher GHQ scores indicated lower level of mental health. Therefore, in multilevel linear models, positive coefficients indicated negative relationships between independent variables and mental health outcomes, while negative coefficients meant positive relationships between independent variables and mental health outcomes. Intra-class correlation coefficients (ICC) were used to estimate the extent to which the neighborhood-level variables accounted for the total variances in GHQ scores. Variance reduction ratios indicated the extent to which the neighborhood-level variables interpreted the variance of mental health in different neighborhoods, thus was used to evaluate the suitability of neighborhood-level variables in models.

## 3. Results

### 3.1. Mental Health and Individual-Level Variables

Table 2 shows the results of five models exploring the effects of the neighborhood environment on mental health. First, the null model showed that the ICC was equal to 21.23%, meaning that 21.23% of the variation in individual mental health came from the neighborhood environment.

Model 1 only included individual-level variables. As shown in Table 2, single, divorced, or widowed respondents reported a significant positive association with GHQ scores, indicating a negative association with mental health (coefficient = 0.79, *p* < 0.1). Education was positively related to mental health. Compared to respondents with technical school or high school education, respondents with junior high school education and below had worse mental health outcomes (higher GHQ scores, coefficient = 0.95, *p* < 0.05) and respondents with education from college, university, and above had better mental health outcomes (lower GHQ scores, coefficient = −0.74, *p* < 0.1). Respondents without housing tenure had worse mental health outcomes than those with housing tenure (coefficient = 1.03, *p* < 0.05). Age, gender, per capita household income per year, per capita living space, and hukou status were not significant covariates with mental health.

### 3.2. Mental Health and Neighborhood Environment

Regarding the effects of the neighborhood-level environment, we used three models with built-environment measured in different buffer areas. In model 2, building density and per capita green area were measured within the road network buffer. Compared to the interclass variance in the null model (1.38), the value reduced to 0.41 in model 2 and the ratio of variance reduction was 70.29%, indicating that 70.29% of the variance of mental health in different neighborhoods was well interpreted by the neighborhood-level variables in model 2.

Model 2 indicated that all built-environment variables of the road network buffer were significantly correlated with mental health. Building density had a positive association with mental health. The coefficient of building density was −0.08 (*p* < 0.01). It indicated when the building density increased one-percentage-point, the GHQ score decreased by 0.08 of a unit. Per capita green area was also positively associated with mental health (coefficient = −0.18, *p* < 0.05). A one-unit increase in the per capita green area decreased the GHQ score by 0.18 units. There was also a significant positive association between neighborhood floor area ratio and mental health (coefficient = −0.56, *p* < 0.01). A one-unit increase in the neighborhood floor area ratio decreased the GHQ score by 0.56 units.

Concerning the neighborhood social capital, neighborhood interaction and neighborhood reciprocity were identified to have significant positive associations with mental health. Specifically, a one-percentage-point increase in the proportion of respondents with high interaction in the neighborhood reduced the GHQ score by 2.86 points (coefficient = −2.86, *p <* 0.1). A one-percentage-point increase in the proportion of respondents with high reciprocity in the neighborhood reduced the GHQ score by 3.25 points (coefficient = −3.25, *p <* 0.1). As for neighborhood safety, mental health was negatively influenced by neighborhood disputes (coefficient = 0.01, *p <* 0.01). A one-unit increase in the number of neighborhood disputes was accompanied by an increase in the GHQ score by 0.01 points.

In model 3, building density and per capita green area were measured within a 1 km circular buffer area. The ratio of variance reduction of model 3 was 52.17%, less than that of model 2. The results of model 3 showed that building density and per capita green area were not statistically significant. Neighborhood floor area ratio had a significant positive effect on mental health (coefficient = −0.75, *p <* 0.01). A one-unit increase in the neighborhood floor area ratio decreased the GHQ score by 0.75 units. As for social environment, there was no evidence to indicate that social capital variables were significantly related to residents’ mental health. Only neighborhood disputes were negatively associated with mental health (coefficient = 0.01, *p <* 0.01). A one-unit increase in the number of neighborhood disputes was accompanied by an increase in the GHQ score by 0.01 points.

In model 4, we tested building density and per capita green area measured within a 500 m circular buffer area. The ratio of variance reduction of model 4 was 50.00%, less than that of model 2 and model 3. Model 4 showed that per capita green area (coefficient = −0.18, *p <* 0.1) and neighborhood floor area ratio (coefficient = −0.77, *p <* 0.01) had positive and statistically significant associations with mental health. A one-unit increase in the per capita green area decreased the GHQ score by 0.18 units, and a one-unit increase in the neighborhood floor area ratio decreased the GHQ score by 0.77 units. Contrary to our expectations, among the four neighborhood social capital variables, only neighborhood participation had a significant negative effect on mental health. A one-percentage-point increase in the proportion of respondents with high participation in the neighborhood was accompanied by an increase in the GHQ score by 4.74 points (coefficient = 4.74, *p <* 0.1). Neighborhood disputes still had a negative impact on mental health (coefficient = 0.01, *p <* 0.01). A one-unit increase in the number of neighborhood disputes increased the GHQ score by 0.01 points.

## 4. Discussion

Existing literature has documented the direct associations between some neighborhood environment attributes and mental health in China, without considering the different effects caused by neighborhood measuring techniques. This study explores the neighborhood determinants of mental health, using different built-environment delineations, and focuses on indicators related to so-called new urbanism or compact cities and contextual social capital, which have not previously been fully investigated.

One of our key findings indicated that built-environment indicators based on road network buffers had a higher explanatory power regarding residents’ mental health outcomes than those based on two circular buffers. This observation confirms that the way in which data are aggregated at the neighborhood level have a significant impact on the relationship between the neighborhood environment and health [62,63]. In Chinese cities, many schools, companies, and gated communities are surrounded by walls and are not accessible to everyone. Therefore, compared to circular buffers, the use of road network buffers can better delineate “true causally relevant” geographic context that people are exposed to, and can better interpret the effect of neighborhoods’ built-environments on mental health.

Neighborhood floor area ratio and building density were positively related to mental health. One possible cause is that compact neighborhoods usually contribute to health-related physical activities [55,64]. This indicates that the concepts of new urbanism and compact city are beneficial to Chinese city dwellers in terms of mental health and can be adopted as a strategy to optimize the urban built-environment. Per capita green area positively affected residents’ mental health, and this result resonated with observations using different greenspace indicators in developed countries and Chinese cities [13,27,65,66]. This observation suggests that green space should be taken into consideration in neighborhood design and city planning.

Comparing the influence of the built-environment based on different buffer zones, only building density extracted from road network buffers had a significant impact on mental health, and per capita green area of road network buffers and 500 m circular buffers were significantly correlated to mental health. This might result from the deviation in the measurement of the built-environment, where inaccessible buildings and greenspace were not supposed to affect residents’ health-related behaviors and mental wellbeing [34]. The different impact of greenspace based on two circular buffer zones may be due to the distance in the measurement. Residents are considered to have more possibilities for exposure to greenspace where they are closer to and exert influence on them [65].

Among social environment variables, only neighborhood disputes showed a consistently significant negative relationship with mental health. Neighborhood interaction and neighborhood reciprocity were only significantly related to mental health in model 2. The reason may lie in the influence of greenspace and building density, as greenspace and high building density can strengthen social capital [6,19,30]. Data derived from road network buffers in model 2 can better reflect the actual green space and building density that residents are exposed to, thus may enhance the effect of social capital on mental health. These findings suggest that harmonious neighborhoods with high neighborhood interaction, high reciprocity, and less disputes are beneficial to mental wellbeing. Practical approaches, such as enriching neighborhood groups and building public space can be adopted to build a cohesive and supportive neighborhood and promote residents’ mental health.

This study has several limitations that need to be addressed. First, as it is based on a cross-sectional dataset, this study is unable to solve the “self-selection” issue. Residents who have some unobserved socio-demographics characteristics (e.g., have higher occupational prestige) tend to choose neighborhoods with better built and social environment, and thereby may report a lower level of GHQ score. Second, this study only investigates the direct relationship between the neighborhood environment and mental health, without further exploring pathways. Built-environment can influence mental health though social capital, physical activity and neighborhood perception [15,21,30]. We plan to test the mediating effect of neighborhood perception in our further studies. Third, there are still some factors that might affect mental health which are not explored in this study and need to be further investigated, such as public service facilities, mixed land use, etc. Forth, the impact of the neighborhood environment on mental wellbeing can vary substantially among demographically and socio-economically different groups [67,68,69]. Moderated analysis of age, gender, income and Hukou status would help to clarify this problem. Finally, the 12-item General Health Questionnaire used in this study assesses respondents’ current mental wellbeing state, and therefore it is more sensitive to short-term mental disorders, but not to long-standing psychiatric attributes.

## 5. Conclusions

This study demonstrates that the neighborhood built-environment and the social environment have significant impacts on residents’ mental health. Neighborhood floor area ratio, building density, and per capita green area are positively correlated with mental health. Neighborhood safety and contextual neighborhood interaction and reciprocity have positive associations with mental health. Our findings also suggest that different delineations of the neighborhood built-environment can influence analytical results concerning the relationship between the neighborhood environment and mental health.

## Figures and Tables

**Table 1:** Summary statistics of residents’ socio-demographics and neighborhood environment.

Variables	Proportion/Mean (S.D.)
GHQ Score	22.63 (5.27)
Gender	
Male	52.26%
Female	47.74%
Age	40.55 (11.07)
Marital status and family organization	
Single, divorced, or widowed	15.31%
Married and living with family	78.17%
Married but not living with family	6.52%
Education	
Junior high school and below	31.82%
Technical school/high school	33.48%
College/university and above	34.70%
Per capita household income per year (10 thousand Yuan)	4.01 (4.31)
Per capita living space (m^2^)	30.78 (21.74)
Housing tenure	
Yes	54.17%
No	45.83%
Hukou status	
Guangzhou hukou holders	59.39%
Non-Guangzhou hukou holders	40.61%
Built-environment	
Building density (network buffer) (%)	37.28 (10.57)
Building density (circular buffer 1 km) (%)	41.82 (11.53)
Building density (circular buffer 500 m) (%)	37.06 (12.00)
Per capita green area (network buffer)	18.68 (19.29)
Per capita green area (circular buffer 1 km)	34.82 (38.12)
Per capita green area (circular buffer 500 m)	24.32 (23.47)
Neighborhood floor area ratio	1.83 (0.89)
Social environment	
Percentage of high interaction	0.31 (0.12)
Percentage of high participation	0.15 (0.11)
Percentage of high reciprocity	0.66 (0.15)
Percentage of high trust	0.56 (0.19)
Neighborhood dispute	22.01 (37.33)

**Table 2:** Multilevel modeling on residents’ mental health in Guangzhou.

Model Predictors	Null Model	Model 1 (only Control Variables)		Model 2 (Road Network Buffer)		Model 3 (Linear Buffer 1 km)		Model 4 (Linear Buffer 500 m)	
	Coeff. (Z Value)	Coeff.	Z Value	Coeff	Z Value	Coeff	Z Value	Coeff.	Z Value
**Controlled variables**									
Gender (ref: male)		0.14	0.46	0.28	0.86	0.28	0.84	0.28	0.86
Age		0.02	1.24	0.03 *	1.68	0.03	1.62	0.03	1.62
Marital status and family organization (ref: married and living with family)									
Single, divorced, or widowed		0.79 *	1.67	0.90	1.49	0.87	1.47	0.86	1.46
Married but not living with family		−0.85	−1.30	−0.55	−0.66	−0.57	−0.68	−0.56	−0.66
Education (ref: Technical school/high school)									
Junior high school and below		0.95 **	2.40	0.69 *	1.71	0.70	1.66	0.68	1.61
College/university and above		−0.74 *	−1.76	−0.76 *	−1.75	−0.69	−1.59	−0.67	−1.54
Per capita household income		0.03	0.68	0.04	1.10	0.04	1.00	0.04	0.97
Per capita living space per year		0.22	1.20	−0.02	−0.09	−0.02	−0.11	−0.02	−0.12
Hukou status (ref: Non−Guangzhou hukou holders)		−0.37	−0.87	−0.56	−1.57	−0.52	−1.42	−0.5	−1.41
Housing tenure (ref: Yes)		1.03 **	2.41	1.57 ***	2.85	1.51 ***	2.73	1.51 ***	2.71
**Neighborhood-level variables**									
Building density				−0.08 ***	−3.18	−0.04	−1.46	−0.03	−1.20
Per capita green area				−0.18 **	−2.11	−0.08	−0.73	−0.18 *	−1.81
Neighborhood floor area ratio				−0.56 ***	−3.00	−0.75 ***	−3.17	−0.77 ***	−3.17
Percentage of high interaction				−2.86 *	−1.69	−2.28	−1.14	−2.29	−1.16
Percentage of high participation				3.19	1.17	4.18	1.41	4.74 *	1.72
Percentage of high reciprocity				−3.25 *	−1.85	−1.79	−0.87	−2.36	−1.21
Percentage of high trust				0.44	0.31	−0.58	−0.33	0.19	0.13
Neighborhood dispute				0.01 ***	3.00	0.01 ***	2.65	0.01 ***	3.37
Constant	10.64 *** (31.79)	10.13 ***	26.76	9.97 ***	22.51	9.98 ***	22.61	9.978 ***	22.78
Interclass variance	1.38	—		0.41		0.66		0.69	
Intra−class variance	5.12	5.17		5.04		5.04		5.04	
Log likelihood	−3447.17	−3523.29		−3416.67		−3420.19		−3420.43	
ICC	21.23%	—		7.52%		11.58%		12.04%	
Variance reduction ratio	—	—		70.29%		52.17%		50.00%	

Note: * *p* < 0.10; ** *p* < 0.05; *** *p* < 0.01.
